# Thyroid function tests in patients at the emergency department compared to a prior healthy setting

**DOI:** 10.1371/journal.pone.0202422

**Published:** 2018-08-20

**Authors:** Rock Bum Kim, Minji Kim, Yoon Young Cho, Soo Kyoung Kim, Jung Hwa Jung, Jaehoon Jung, Chi Yeon Kim, Dawon Kang, Jong Ryeal Hahm

**Affiliations:** 1 Regional Cardiocerebrovascular Disease Center, Gyeongsang National University Hospital, Jinju, Korea; 2 Department of Endocrinology and Metabolism, Department of Internal Medicine, Gyeongsang National University Hospital, Jinju, Korea; 3 Institute of Health Sciences, Gyeongsang National University College of Medicine, Jinju, Korea; 4 Department of Internal Medicine, Gyeongsang National University Changwon Hospital, Changwon, Korea; 5 Department of Dermatology, College of Medicine, Gyeongsang National University, Jinju, Korea; 6 Department of Physiology, College of Medicine, Gyeongsang National University, Jinju, Korea; 7 Department of Internal Medicine, College of Medicine, Gyeongsang National University, Jinju, Korea; University of California Los Angeles, UNITED STATES

## Abstract

We examined the changes in thyroid hormone levels in patients with an acute clinical condition and compared these to levels in the healthy subjects. Serum total triiodothyronine (T3), thyroid stimulating hormone (TSH), and free thyroxine (fT4) measurements were recorded from 555 patients (mean age: 55.0 years, men: 65.9%) admitted to the emergency department (ED) 1–91 months (median: 34 months) after a regular health examination (HE). Serological data were analyzed; mean change in hormone levels was stratified by emergency classification system and quintiles of changes in inflammatory marker values, such as neutrophil lymphocyte ratio (NLR) and high-sensitivity C-reactive protein (CRP). The mean decrease in T3 levels from HE and ED samples was 10.6 ng/dL (*p*< 0.001). Mean decrease in T3 levels was 21.6 ng/dL among patients classified as having an infection status and 11.0 ng/dL among patients classified as having an urgency status. A decrease 3.7 ng/dL among emergency patients was observed. TSH and fT4 levels did not change across all groups. When patients were stratified into quintiles according to changes in NLR values, mean decreases in T3 were 6.21, 8.14, 14.37, 12.76, and 21.98 ng/dL and showed significant linear reduction (*p*<0.001). For quintiles of changed CRP values, mean decreased T3 levels were 10.57, 3.05, 4.47, 7.68, and 28.07 ng/dL. TSH and fT4 were not associated with significant changes (*p* = 0.100, *p* = 0.561, respectively). In this study, thyroid function changes in individuals with an acute condition revealed that T3 significantly decreased, more markedly in infectious diseases compared to their healthy counterparts, and decline in T3 measurements correlated with inflammatory markers. TSH and fT4 levels remained stable. It is necessary to consider the severity of acute conditions when abnormal T3 levels are detected in subjects with emergent status.

## Introduction

Thyroid hormone changes are a common feature in emergency patients with no known thyroid dysfunction. A decrease in triiodothyronine (T3) is frequently observed with normal levels of free thyroxine (fT4) and thyroid-stimulating hormone (TSH), a condition known as euthyroid sick syndrome (ESS), non-thyroidal illness syndrome (NTIS), or low T3 syndrome[1]. Thyroid hormone changes are believed to be physiological adaptations owing to re-allocation and metabolism of body energy caused by an acute illness[2]. Therefore, in many cases, thyroid hormone levels return to normal once the illness or stress condition is overcome[3]. Accordingly, therapy for deranged thyroid hormone levels is generally not initiated in patients with NTIS, and the benefits of treatment are unclear[4].

NTIS frequently occurs in patients with acute/severe illness admitted to the intensive care unit (ICU) or emergency department (ED). Several studies have reported thyroid hormone changes in patients with sepsis, burns, acute ischemic syndromes, physical trauma, and bone marrow transplantation, as well as in those who undergo major surgery, such as coronary-artery bypass surgery[2,5–12]. Furthermore, changes in thyroid hormone levels have been associated with starvation and have been reported in premature infants[3]. However, most studies have reported hormonal changes occurring between disease onset and treatment and do not provide data on hormone changes during the healthy state prior to disease onset. Moreover, because studies have reported hormonal changes in patients with specific diseases (e.g., acute myocardial infarction, sepsis), it is difficult to compare the differences in thyroid hormones level changes according to the severity of the patient’s condition or the clinical classification of patient status in the ICU or ED.

This study examines the extent of changes in thyroid hormone levels among previously healthy individuals presenting as emergencies. Furthermore, thyroid hormone changes are analyzed according to emergency condition type (emergency status, urgency status, and infection). In addition, we examine the association between the degree of change in the inflammatory markers and thyroid hormones.

## Methods

### Study participants

A total of 57,310 healthy individuals undergoing health examinations (HEs) in a tertiary hospital with 900 beds between January 2007 and December 2015 were identified by a search of electronic medical records (EMR). Patients with abnormal TSH or fT4 levels and those with a previous diagnosis of thyroid disease, such as Graves’ disease or Hashimoto’s thyroiditis, were excluded. Finally, 555 patients treated at the hospital ED were included in this study. The median time from HE to ED visit was 34 months (range, 1–91 months). The frequency of their HEs during the study period was mean 2.15 (SD 1.82) and median 1.0 (range, 1.0–9.0).

This study was approved by the Institutional Review Board (IRB 2016-02-015) of Gyeongsang National University Hospital and was conducted in accordance with the Declaration of Helsinki. The study was retrospectively conducted, and the requirement of informed consent for the study was exempt due to restrained database access for analysis purposes only.

### Thyroid hormone and laboratory measurements

Results of clinical tests from HEs and on ED visit were obtained from EMR. For patients with multiple HEs prior to ED visit, the mean value of the laboratory measurements was used. The following parameters were measured: thyroid hormones, such as T3, TSH, and fT4 using chemistry luminescence immunoassay with the same company equipment (Cobas series, Roche Diagnostic, Switzerland) and inflammation markers, such as white blood cells (WBC), erythrocyte sedimentation rate (ESR), neutrophil lymphocyte ratio (NLR), high-sensitivity C-reactive protein (CRP), serum red blood cell (RBC), hemoglobin, hematocrit, platelets, glucose, hemoglobin A1c (HbA1c), blood urea nitrogen (BUN), creatinine (Cr), total cholesterol, triglyceride, low density lipid (LDL), high density lipid (HDL), alanine aminotransferase (ALP), aspartate aminotransferase (AST), alkaline phosphatase (ALP), albumin, protein, creatinine kinase (CK), and lactate dehydrogenase (LDH).

### Classification of emergency patients

Patient ED records, laboratory results, and treatment were reviewed using EMR, and diagnosis at the ED of each patient was listed as the common reason for the visit. Furthermore, patient status was classified into infection, emergency, and urgency (non-emergency) conditions, according to the Canadian Triage and Acuity Scale (CTAS) adult guidelines[13]. The CTAS guidelines are a triage system that classifies patients admitted to ED into five classes (resuscitation, emergent, urgent, less urgent, and non-urgent) on the basis of patient symptoms, vital signs, initial laboratory results, and examination findings. In the present study, classes 1 and 2 were defined as emergency classes, while classes 3, 4, and 5 were defined as urgency classes. Infection was defined as body temperature of >39°C with positive bacterial cultures.

### Quintiles of categorization of inflammatory markers changed value

To examine the association between the changes in inflammatory markers and thyroid hormones, ESR, WBC, NLR, and CRP changes were computed. Patients whose inflammatory markers decreased farther from that at the time of the ED visit were considered to have no inflammation and were excluded from the analysis. Patients with increased inflammatory markers on ED admission relative to HE levels were classified into quintiles. The quintile values for degree of changed ESR(mm/h) were as follows: 1st quintile(Q1): 0.00 to <2.00, 2nd quintile(Q2): 2.00 to <6.00, 3rd quintile(Q3): 6.00 to <14.70, 4th quintile(Q4): 14.70 to <30.12, 5th quintile(Q5): ≥30.12. The quintile values for degree of changed WBCs (10^3^ cells/ mm^3^) were as follows: Q1: 0.00 to <0.75, Q2: 0.75 to <1.56, Q3: 1.56 to <2.58, Q4: 2.58 to <4.58, Q5: ≥4.58. The quintile values for degree of changed NLR were as follows: Q1: 0.00 to <0.32, Q2: 0.32 to <0.77, Q3: 0.77 to <1.74, Q4: 1.74 to <3.86, Q5: ≥3.86. The quintile values for degree of changed CRP(mg/L) were as follows: Q1: 0.00 to <0.10, Q2: 0.10 to <0.45, Q3: 0.45 to <2.07, Q4: 2.07 to <20.02, Q5: ≥20.02.

### Statistical analysis

All continuous results are presented as mean values and standard deviation. The differences in thyroid hormone levels and laboratory measurement results during HE and ED visit were analyzed. Variables satisfying the homogeneity of the variance test were analyzed using the paired t-test and those that did not satisfy the homogeneity of variance test were analyzed using the Wilcoxon signed-rank test. Differences in test results between HE and ED visit were analyzed for each of the three emergency classes. Furthermore, the average thyroid hormone changes for each of the three emergency classes were compared using the Kruskal–Wallis test. Proportions of patients who had abnormal thyroid hormone levels at ED visit were calculated and compared between three emergency classes using Fisher’s exact test.

To examine the association between changes in inflammatory markers (ESR, WBC, NLR, and CRP) and changes in thyroid hormone levels, differences in thyroid hormone changes according to quintiles of inflammatory changes were analyzed using the Kruskal–Wallis test. Furthermore, trends of linear increase or decrease in hormone levels according to quintiles were analyzed using the Jonckheere–Terpstra trend test.

A *p-*value of <0.05 was considered statistically significant for two-tailed tests. IBM SPSS Statistics for Windows (version 20.0. Armonk, NY: IBM Corp.) was used for all statistical analyses.

## Results

### Difference between healthy examination and emergency department laboratory test results

A total of 555 patients underwent a HE and subsequently visited an ED for treatment. Overall, 65.9% of patients were men, and the mean age at the time of HE was 55.0 (±11.5) years. The median time from HE to ED visit was 34 months (1–91 months). Table 1 presents the laboratory test results at HE and ED visit and the differences between the two results. There were differences in the majority of test results between the two time points. WBC, ESR, CRP, NLR, glucose, HbA1c, AST, ALT, CK-MB, and LDH were increased on ED visit, while RBC, hemoglobin, hematocrit, platelet count, total cholesterol, HDL, and LDL cholesterol were decreased. There were no significant changes in ALP and BUN between the two measurements. T3 decreased by 10.6 ng/dL, from an average of 111.0 ng/dL to 100.4 ng/dL (*p*<0.001). However, mean TSH and fT4 levels did not significantly differ between the two time points (*p* = 0.204, *p* = 0.929, respectively).

**Table 1:** Participant characteristics and laboratory test results at the health examination and on emergency department admission.

	N	Health examination, mean (±SD)	Emergency Department, mean (±SD)	*p*-value
Male, %	555	65.9%		
Age, years	555	55.0 (±11.5)		
WBC, 10^3^cells/ mm^3^	555	6.3 (±1.9)	8.3 (±3.6)	<0.001
RBC, 10^6^ cells/ mm^3^	555	4.7 (±0.5)	4.5 (±0.6)	<0.001
Hemoglobin, g/dL	555	14.4 (±1.6)	13.8 (±1.8)	<0.001
Hematocrit, %	555	42.8 (±4.4)	40.4 (±5.0)	<0.001
Platelet, 10^3^ cells/ mm^3^	555	255.9 (±66.5)	240.4 (±71.0)	<0.001
ESR, mm/h	291	14.5 (±13.5)	18.2 (±21.2)	0.001
Hs-CRP, mg/L	398	2.1 (±10.0)	12.2 (±37.5)	<0.001
NLR	555	1.7 (±1.0)	3.2 (±3.8)	<0.001
Glucose, mg/dL	300	93.3 (±23.3)	141.0 (±63.5)	<0.001
HbA1c, %	357	5.9 (±0.9)	6.0 (±1.2)	0.004
Total cholesterol, mg/dL	349	192.1 (±35.1)	176.0 (±39.8)	<0.001
Triglyceride, mg/dL	413	141.2 (±88.2)	154.6 (±133.7)	0.031
HDL cholesterol, mg/dL	402	51.6 (±14.1)	48.4 (±14.6)	<0.001
LDL cholesterol, mg/dL	402	120.9 (±33.1)	113.1 (±37.5)	<0.001
ALP, U/L	301	67.5 (±18.3)	78.3 (±104.3)	0.067
AST, U/L	301	25.8 (±13.5)	40.4 (±60.1)	<0.001
ALT, U/L	301	26.2 (±18.6)	34.3 (±50.1)	0.005
BUN, mg/dL	300	15.1 (±4.7)	15.6 (±7.3)	0.155
Creatinine, mg/dL	299	0.8 (±0.2)	0.9 (±0.6)	0.004
CK-MB, ng/mL	252	117.8 (±125.8)	240.1 (±712.1)	0.008
LDH, u/L	257	184.2 (±35.0)	243.6 (±297.8)	0.001
T3, ng/dL	517	111.0 (±18.7)	100.4 (±23.7)	<0.001
TSH, mlU/L	524	2.1 (±1.3)	2.2 (±1.8)	0.204
fT4,ng/dL	517	1.3 (±0.2)	1.3 (±0.3)	0.929

Abbreviations: WBC, white blood cell; RBC, red blood cell; ESR, erythrocyte sedimentation rate; NLR, neutrophil to lymphocyte ratio; CRP, C-reactive protein; HbA1c, glycated hemoglobin; HDL, high density lipoprotein; LDL, low density lipoprotein; ALP, alkaline phosphatase; AST, aspartate aminotransferase; ALT, alanine aminotransferase; BUN, blood urea nitrogen; CK-MB, creatine kinase-muscle/brain; LDH, lactate dehydrogenase; T3, triiodothyronine; TSH, thyrotropin; fT4, free thyroxine.

### Difference between laboratory test results at the health examination and on emergency department admission by classification

The common disease among infectious patients was pulmonary infectious disease, such as pneumonia or bronchitis. Coronary artery disease required emergency percutaneous coronary intervention and was the most common disease in emergency patients. In urgency patients, heart-related symptoms and diagnosis were most common (S1 Table).

**Fig 1:**
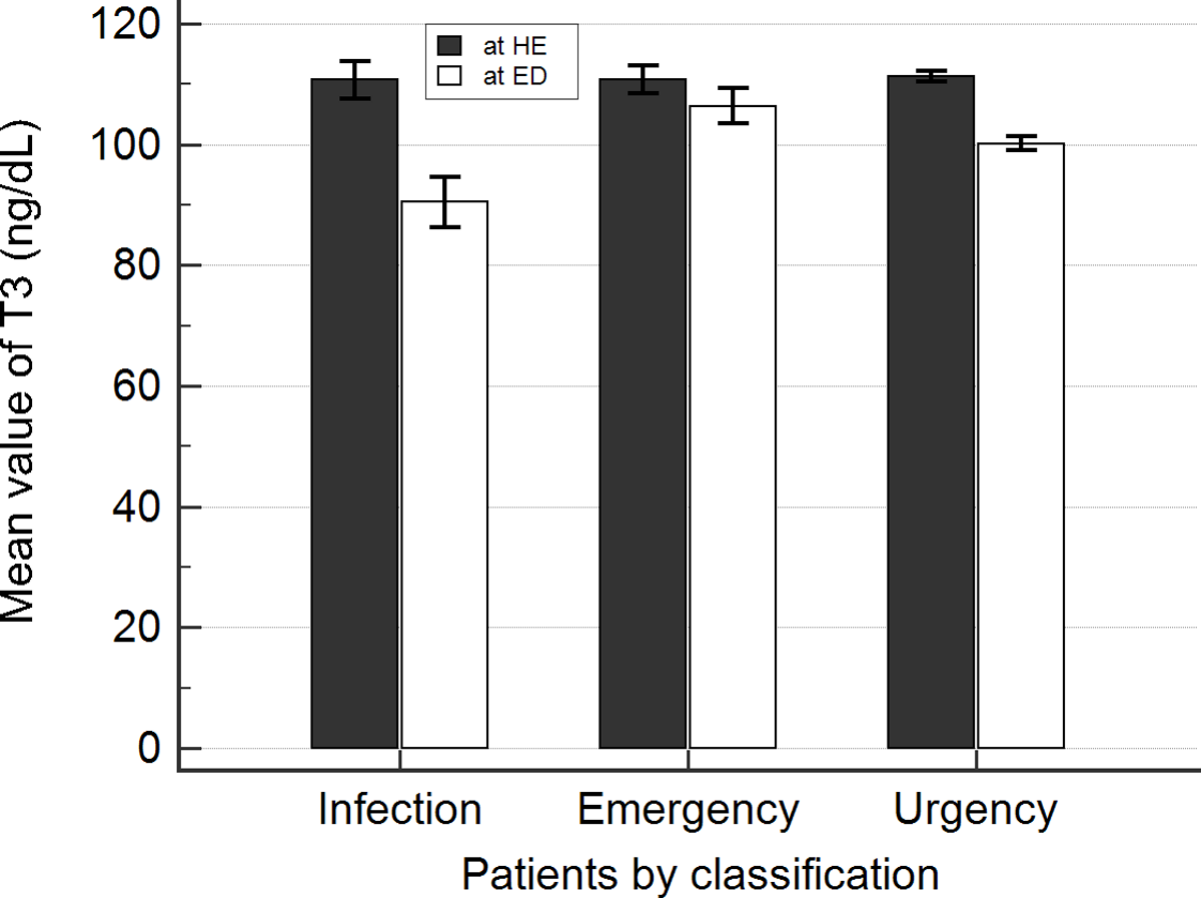
Mean value of T3 levels at health examination and emergency department admission according to classification. In infection patients (N = 32) and urgency class patients (N = 460), significantly decreased T3 levels were observed at ED compared to that at HEs; however, this was not decreased in emergency class patients (N = 63). Error bars indicate ±1 standard errors. HE = health examination, ED = emergency department.

Patients presenting to ED were classified into three groups to compare the changes indifferent clinical laboratory test results and thyroid hormones. Changes in many clinical indicators (e.g., WBC, RBC, hemoglobin, hematocrit, CRP, and NLR) were observed for patients with infection (N = 32). T3 decreased by 21.6 ng/dL between HE and ED visit (*p*< 0.001). However, TSH and fT4 remained stable (*p-*value 0.966 and 0.512, respectively). Sixty-three patients were classified into the emergency class and these patients showed almost no changes in the three thyroid hormone levels (*p*> 0.05). The urgency class comprised 460 patients and T3 levels decreased by 11.0 ng/dL (*p*<0.001) in these patients, although only minor changes in TSH and fT4 levels (*p-*value 0.298 and 0.800, respectively) were observed (Table 2 and Fig 1).

**Table 2:** Means of difference between health status and emergency disease status by classification.

	Infection patients (N = 32)		Emergency class patients (N = 63)		Urgency class patients (N = 460)	
	Mean Difference	*p*-value	Mean Difference	*p*-value	Mean Difference	*p*-value
WBC, 10^3^cells/ mm^3^	3.9	< 0.001	3.9	< 0.001	1.5	< 0.001
RBC, 10^6^ cells/ mm^3^	-0.5	< 0.001	-0.1	0.100	-0.2	< 0.001
Hemoglobin, g/dL	-1.8	< 0.001	-0.2	0.470	-0.5	< 0.001
Hematocrit, %	-5.7	< 0.001	-1.5	0.013	-2.4	< 0.001
Platelet, 10^3^ cells/ mm^3^	-42.1	0.006	-10.8	0.029	-14.3	< 0.001
ESR, mm/h	34.0	< 0.001	-1.5	0.268	0.8	0.464
Hs-CRP, mg/L	75.7	< 0.001	4.2	0.010	6.7	< 0.001
NLR	5.5	< 0.001	1.5	< 0.001	1.2	< 0.001
Glucose, mg/dL	51.5	< 0.001	71.5	< 0.001	44.1	< 0.001
HbA1c, %	0.4	0.186	0.1	0.455	0.1	0.015
Total cholesterol, mg/dL	-40.3	0.001	-8.9	0.180	-15.3	< 0.001
Triglyceride, mg/dL	-18.8	0.188	9.3	0.433	15.5	0.032
HDL cholesterol, mg/dL	-1.7	0.533	-1.5	0.220	-3.6	< 0.001
LDL cholesterol, mg/dL	-31.0	0.003	1.6	0.807	-8.2	< 0.001
ALP, U/L	14.4	0.261	4.6	0.445	11.2	0.114
AST, U/L	11.4	0.058	32.7	0.006	12.5	< 0.001
ALT, U/L	6.7	0.571	12.2	0.045	7.6	0.023
BUN, mg/dL	2.6	0.290	-0.2	0.537	0.4	0.282
Creatinine, mg/dL	0.1	0.373	0.1	0.001	0.1	0.023
CK-MB, ng/mL	47.5	0.923	132.6	0.008	126.2	0.021
LDH, u/L	110.2	0.008	63.5	0.001	55.0	0.013
T3, ng/dL	-21.6	< 0.001	-3.7	0.155	-11.0	< 0.001
TSH, mlU/L	-0.1	0.966	0.3	0.639	0.1	0.298
fT4,ng/dL	0.0	0.512	0.0	0.950	0.0	0.800

Abbreviations: WBC, white blood cell; RBC, red blood cell; ESR, erythrocyte sedimentation rate; NLR, neutrophil to lymphocyte ratio; CRP, C-reactive protein; HbA1c, glycated hemoglobin; HDL, high density lipoprotein; LDL, low density lipoprotein; ALP, alkaline phosphatase; AST, aspartate aminotransferase; ALT, alanine aminotransferase; BUN, blood urea nitrogen; CK-MB, creatine kinase-muscle/brain; LDH, lactate dehydrogenase; T3, triiodothyronine; TSH, thyrotropin; fT4, free thyroxine.

Proportions of patients with decreased levels from the normal range of T3, TSH, and fT4 at ED visit were 17.2%, 2.3%, and 3.1%, respectively, while the proportions of patients with increased levels were 0.4%, 11.5%, 4.2%, respectively. There were no statistical differences in the proportions between the three emergency classes (all *p*> 0.05) (Table 3).

**Table 3:** Numbers and proportions of patients who had abnormal thyroid hormone levels by three emergency classes.

	Total patients, N (%)	Infection patients, N (%)	Emergency class patients, N (%)	Urgency class patients, N (%)	*p*-value
T3					
Decreased from normal	89 (17.2)	9 (36.0)	7 (11.7)	73 (16.9)	0.101
Normal range (80 ~ 200 ng/dL)	426 (82.4)	16 (64.0)	53 (88.3)	357 (82.6)	
Increased from normal	2 (0.4)	0 (0.0)	0 (0.0)	2 (0.5)	
TSH					
Decreased from normal	12 (2.3)	1 (4.0)	1 (1.6)	10 (2.3)	0.738
Normal range (0.27 ~ 4.2 mlU/L)	452 (86.3)	20 (80.0)	54 (88.5)	378 (86.3)	
Increased from normal	60 (11.5)	4 (16.0)	6 (9.8)	50 (11.4)	
fT4					
Decreased from normal	16 (3.1)	2 (8.0)	2 (3.3)	12 (2.8)	0.262
Normal range (0.93 ~ 1.7 ng/dL)	484 (92.7)	21 (84.0)	56 (91.8)	407 (93.3)	
Increased from normal	22 (4.2)	2 (8.0)	3 (4.9)	17 (3.9)	

Abbreviations: T3, triiodothyronine; TSH, thyrotropin; fT4, free thyroxine.

### Association between inflammatory markers and thyroid hormones

**Fig 2:**
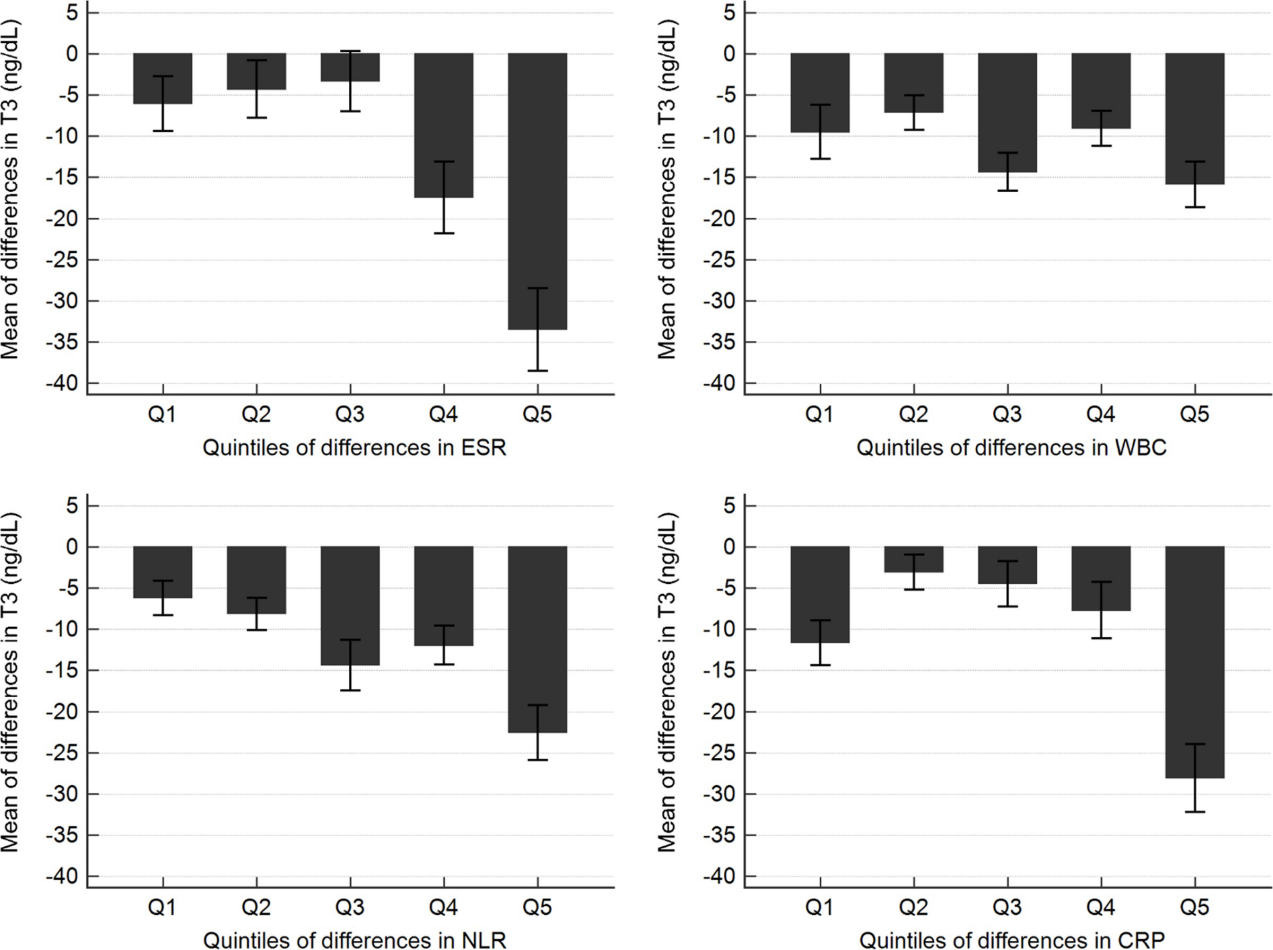
Means of difference in T3 levels according to quintiles of different inflammatory markers between health status and emergency disease status. The error bars indicate ±1 standard errors. The negative values of T3 levels represents the decrease in the emergency disease status compared to health status. Quintile values of differences in ESR are as follows: 0.00≤Q1<2.00, 2.00≤Q2<6.00, 6.00≤Q3<14.70, 14.70≤Q4<30.12, 30.12≤Q5. Differences in WBC are as follows: 0.00≤Q1< 0.75, 0.75≤Q2<1.56, 1.56≤Q3<2.58, 2.58≤Q4<4.58, 4.58≤Q5. Differences in NLR are as follows: 0.00≤Q1<0.32, 0.32≤Q2<0.77, 0.77≤Q3<1.74, 1.74≤Q4<3.86, 3.86≤Q5. Differences in CRP are as follows: 0.00≤Q1<0.10, 0.10≤Q2<0.45, 0.45≤Q3<2.07, 2.07≤Q4<20.02, 20.02≤Q5.

The association between inflammatory markers and thyroid hormones was analyzed by calculating the changes in the inflammatory markers and classifying these into quintiles. Results showed that T3 levels varied according to ESR, NLR, and CRP quintiles but not WBC quintiles. The greatest reduction in T3 levels was observed in Q5, which was the quintile associated with the greatest change in inflammatory markers. T3 levels decreased by 33.50 ng/dL in ESR Q5, by 21.98 ng/dL in NLR Q5, and by 28.07 ng/dL in CRP Q5 (Table 4 and Fig 2). In addition, the reduction of T3 linearly increased with increasing quintiles for ESR, NLR, and CRP (*p* for trend <0.001). There were no significant changes in TSH and fT4 according to quintiles of all inflammatory markers.

**Table 4:** Means of differences in thyroid hormone levels between health status and emergency disease status according to quintiles of differences in inflammatory markers.

		Difference in T3, Mean (±SD)	*p*-value	Difference in TSH, Mean (±SD)	*p*-value	Difference in fT4, Mean (±SD)	*p*-value
ESR (N = 124)	Q1	-6.05 (±15.61)	<0.001^*^	0.01 (±1.48)	0.913^*^	0.06 (±0.20)	0.236^*^
	Q2	-4.31 (±17.12)	<0.001^†^	0.01 (±1.77)	0.338^†^	0.11 (±0.37)	0.030^†^
	Q3	-3.30 (±20.37)		0.10 (±1.76)		-0.01 (±0.19)	
	Q4	-17.45 (±20.46)		-0.08 (±2.90)		-0.05 (±0.25)	
	Q5	-33.50 (±25.18)		-0.28 (±2.64)		-0.07 (±0.34)	
WBC (N = 399)	Q1	-9.51 (±29.09)	0.080^*^	0.01 (±1.95)	0.284^*^	-0.01 (±0.24)	0.889^*^
	Q2	-7.13 (±19.13)	0.257^†^	-0.07 (±1.43)	0.787^†^	-0.01 (±0.18)	0.797^†^
	Q3	-14.11 (±19.21)		0.30 (±2.08)		0.02 (±0.29)	
	Q4	-8.95 (±19.54)		0.38 (±1.88)		-0.05 (±0.26)	
	Q5	-15.87 (±24.04)		0.01 (±1.63)		0.03 (±0.34)	
NLR (N = 339)	Q1	-6.21 (±17.93)	<0.001^*^	0.18 (±1.57)	0.209^*^	-0.01 (±0.21)	0.421^*^
	Q2	-8.14 (±16.60)	<0.001^†^	-0.27 (±1.55)	0.054^†^	-0.03 (±0.19)	0.310^†^
	Q3	-14.37 (±24.65)		-0.02 (±1.45)		0.01 (±0.21)	
	Q4	-12.76 (±20.35)		-0.07 (±1.60)	-0.01 (±0.33)	
	Q5	-21.98 (±26.13)		-0.41 (±1.93)		0.06 (±0.36)	
CRP (N = 200)	Q1	-10.57 (±13.99)	<0.001^*^	0.00 (±1.99)	0.100^*^	0.01 (±0.21)	0.561^*^
	Q2	-3.05 (±13.78)	<0.001^†^	0.21 (±1.16)	0.375^†^	0.00 (±0.19)	0.227^†^
	Q3	-4.47 (±18.73)		0.71 (±1.67)		-0.03 (±0.19)	
	Q4	-7.68 (±22.87)		-0.29 (±1.55)		0.00 (±0.20)	
	Q5	-28.07 (±26.80)		-0.07 (±2.46)		-0.09 (±0.32)	

* The p-values mean the difference between the medians value of quintile groups resulted by Kruskal-Wallis test.

† The p-values mean the order trend of medians for quintile groups resulted by Jonckheere’s trend test.

Abbreviations: ESR, erythrocyte sedimentation rate; WBC, white blood cell; NLR, neutrophil to lymphocyte ratio; CRP, C-reactive protein; T3, triiodothyronine; TSH, thyrotropin; fT4, free thyroxine.

## Discussion

This study examined changes in thyroid hormones associated with infection, emergency, or urgency conditions among previously healthy individuals (healthy state determined on the basis of previous HEs). Furthermore, the extent of hormonal changes in relation to changes in inflammatory markers was analyzed. Results demonstrated that thyroid hormone levels changed with the onset of an emergency condition in previously healthy individuals. Among different thyroid hormones, T3 decreased by approximately 10 ng/dL, although small or no changes were observed in TSH and T4 levels. The greatest T3 reduction was observed among individuals who developed an infection, followed by individuals in an urgency condition. TSH and fT4 levels remained largely unchanged across the groups. When inflammatory markers (e.g., ESR, NLR, CRP) were divided into quintiles, T3 reduction positively correlated with changes in inflammatory markers.

Comparable findings have been reported in studies involving patients presenting to ED with acute illness; these studies reported that the greatest T3 reduction was observed among patients requiring emergency surgery or trauma patients[7,14–16], patients with acute coronary artery disease or acute heart failure[2,9,17–19], acute cerebrovascular disease[20–23], and acute infection or sepsis[24–26]. These findings corroborate our results and are consistent with decreases in T3 levels reported to be associated with fasting, stress, acute illness, and chronic illness. Most studies identified the percentage of patients with thyroid abnormalities but did not examine the average decrease in thyroid hormones, as reported in our study. Furthermore, a major limitation of these studies is that the extent of the decrease in thyroid hormone levels cannot be determined, because data in the healthy state were not collected; therefore, these studies simply conclude that thyroid hormone levels decreased since the onset of illness. The difference between previous studies and this longitudinal observation study is that our study quantified thyroid hormone changes from the healthy state to the onset of acute illness.

TSH and fT4 levels did not change significantly. Although TSH levels generally remain unchanged in NTIS[1], fT4 changes have been previously reported[16,27]. However, fT4 levels may not change in the early stages of illness, because the reduction may vary according to disease severity and its long half-life of approximately 1 week[1]. We noted that fT4 levels have been reported to increase in the early stages after heart surgery, decreasing 12 hour later[27]. A study further reported decreased fT4 levels 48 hour following an injury[16]. Therefore, future studies should consider the severity of the disease and the time since disease onset should be considered when interpreting the lack of changes in fT4 levels in the present study. This may be attributed to the fact that patients in the urgency class, representing a less severe status, account for 82.9% (N = 460) of the entire subject pool and that results of tests conducted on admitted patients following ED visit were not collected.

This study classified patients with acute conditions into three classes (infection, emergency, urgency) and examined the changes in thyroid hormone levels. Thyroid hormones, particularly T3, did not decrease in all acute illnesses. The greatest decrease in T3 levels relative to normal levels (HE examination levels) were observed in patients presenting to ED with an infectious disease, followed by patients in the urgency class. No decrease in T3 levels was observed among patients with emergency conditions, such as myocardial infarction. These results are in contrast with previous findings. However, T3 is known to gradually decrease over time after disease onset[1] and reach the lowest level a few days or even a few weeks after disease onset[9,27–29]; thus, we cannot conclude with certainty that T3 levels do not decrease in emergency conditions. Patients with acute illnesses tend to visit the ED promptly; therefore, the time from disease onset to testing is short. There is a possibility that the decrease in T3 levels may have been small at the time of testing but may subsequently increase. To confirm this hypothesis, T3 levels should be monitored in the ward following admission; however, these data could not be collected in our study. Conversely, the finding that patients with infectious diseases exhibited the greatest decrease in T3 levels may be attributed to the fact that this category of patients tends to visit the hospital a few days after symptom onset (e.g., fever, general weakness).

One of the important findings of this study was that changes in the inflammatory markers were associated with T3 level changes, with decreases in T3 associated with greater changes in inflammatory markers. In particular, T3 reduction was strongly associated with decreases in NLR, ESR, and CRP. The marked changes in T3 levels associated with inflammatory diseases are known to result from cytokine secretion, which is increased in infection and hinders the regulation of the hypothalamus pituitary thyroid axis[16,30]. Type 3 deiodinase is highly expressed as a result of infection and facilitates the conversion of T4 into reverse T3 and the conversion of biologically active T3 to biologically inactive T2, thereby decreasing T3 levels[31]. The association between changes in inflammatory markers and decreased in T3 levels appear to reflect this mechanism. Bacteria population was negatively correlated with T3 levels in an animal study, with increases in bacteria population with decreasing T3 levels[31]. Lee et al.[26] reported that T3 concentration was negatively correlated with CRP concentration, although it was not associated with ESR. Few studies have investigated the association between T3 and other inflammatory markers, such as NLR, ESR, and CRP. The present study is important because it contributes to the existing literature by examining this association in detail.

This longitudinal observation study, reviewed participant test results from a healthy state until disease onset. However, a few limitations should be noted. First, this study did not investigate duration from onset of symptoms to thyroid function testing. Since we did not collect information regarding the time of symptom onset and the time of ED arrival, we could not confirm at which point in time from symptom onset T3 levels began to decrease. Second, we could not review the clinical outcomes after the decrease in thyroid hormone levels in individuals with NTIS. As a result, we could not examine the major clinical outcomes, such as mortality and complication rates associated with the reduction in thyroid hormone levels. Finally, various drugs such as lithium, steroid hormones, amiodarone, aminoglutethimide, and iodine-containing medications can influence the thyroid function test[32]. However, in this study setting, we did not investigate the use of these drugs, because the participants of our study were subjects who had undergone general HEs and ED visits, and the study purpose was examination of the changes of TH by inflammatory markers. Therefore, our result may misinterpret the conclusion because of this limitation. However, we believe that the number of drug uses that can have a critical influence on results is small based on our clinical experience.

In conclusion, this study confirmed that T3 levels decreased among patients who developed an acute illness from a previously healthy state. When the patients were classified into infection, emergency, and urgency classes, T3 significantly decreased among patients in the infection and urgency classes. However, only small decreases in T3 were observed among patients categorized as emergency class and these were not statistically significant. There were no changes in TSH and fT4 levels across the three groups. Increases in inflammatory markers (ESR, NLR, and CRP), with the exception of WBC, were associated with decreases in T3levels.

## Supporting information

S1 Table Diagnosis at the emergency department by classification in study patients. (DOCX)
