# Supplementary material for: Thyroid function tests in patients at the emergency department compared to a prior healthy setting
Source: PLoS One. 2018 Aug 20;13(8):e0202422. doi: 10.1371/journal.pone.0202422 (PMC6101387; doi:10.1371/journal.pone.0202422)
Supplement: S1 Table — (DOCX) [file pone.0202422.s001.docx]

Supplementary Table 1. Diagnosis at the emergency department by classification in study patients.

| **Classification** | **Diagnosis at emergency department** | **N** | **%** |
| --- | --- | --- | --- |
| Infection patients (N=32) | Pneumonia or Bronchitis | 12 | 37.5% |
|  | Upper or other respiratory tract infection | 4 | 12.5% |
|  | Biliary tract or intestinal infection | 4 | 12.5% |
|  | Urinary tract infection | 2 | 6.3% |
|  | Scrub typhus | 5 | 15.6% |
|  | viral meningitis | 1 | 3.1% |
|  | pericardial abscess | 1 | 3.1% |
|  | perianal abscess | 1 | 3.1% |
|  | fever of unknown origin | 2 | 6.3% |
| Emergency class patients (N=63) | Coronary artery disease needed emergency PCI | 35 | 55.6% |
|  | Emergency arrhythmia | 11 | 17.5% |
|  | Cerebrovascular infarction or hemorrhage | 10 | 15.9% |
|  | Aortic dissection | 2 | 3.2% |
|  | Bleeding esophageal varices | 1 | 1.6% |
|  | Pulmonary thromboendarterectomy | 1 | 1.6% |
|  | Hematemesis with pancreatic cancer | 1 | 1.6% |
|  | Thrombotic Thrombocytopenic Purpura/hemolytic uremic syndrome with arrest | 1 | 1.6% |
|  | Dug(imidaclprid) intoxication | 1 | 1.6% |
| Urgency class patients (N=460) | Coronary artery disease needed not PCI | 94 | 20.4% |
|  | Arrhythmia | 47 | 10.2% |
|  | Non-specific chest pain & discomfort | 92 | 20.0% |
|  | Syncope | 29 | 6.3% |
|  | hyperventilation syndrome | 6 | 1.3% |
|  | Pulmonary disease | 9 | 2.0% |
|  | Gastroesophageal reflux disease | 10 | 2.2% |
|  | Abdominal / intestinal disease | 27 | 5.9% |
|  | Central never system disorder | 26 | 5.7% |
|  | Ophthalmic disease | 6 | 1.3% |
|  | Traumatic fracture | 5 | 1.1% |
|  | Musculoskeletal disease | 11 | 2.4% |
|  | Drug intoxication | 16 | 3.5% |
|  | Psychotic disorders | 19 | 4.1% |
|  | Cancer related complications | 11 | 2.4% |
|  | Other disease or Not diagnostic disease | 52 | 11.3% |
